# Modifying an Active Compound’s Release Kinetic Using a Supercritical Impregnation Process to Incorporate an Active Agent into PLA Electrospun Mats

**DOI:** 10.3390/polym10050479

**Published:** 2018-04-27

**Authors:** Carol López de Dicastillo, Carolina Villegas, Luan Garrido, Karina Roa, Alejandra Torres, María José Galotto, Adrián Rojas, Julio Romero

**Affiliations:** 1 Food Packaging Laboratory (Laben), Department of Science and Food Technology, Faculty of Technology, Center for the Development of Nanoscience and Nanotechnology (CEDENNA), Universidad de Santiago de Chile (USACH), 9170201 Santiago, Chile; carolina.villegasvallejos@gmail.com (C.V.); luan.garrido@usach.cl (L.G.); karina.roa@usach.cl (K.R.); alejandra.torresm@usach.cl (A.T.); maria.galotto@usach.cl (M.J.G.); 2 Laboratory of Membrane Separation Processes (LabProSeM), Department of Chemical Engineering, Engineering Faculty, University of Santiago de Chile (USACH), 9170201 Santiago, Chile; adrian.rojass@usach.cl (A.R.); julio.romero@usach.cl (J.R.)

**Keywords:** electrospinning, poly (acid lactic), cinnamaldehyde, supercritical impregnation, release kinetic

## Abstract

The main objective of this work was to study the release of cinnamaldehyde (CIN) from electrospun poly lactic acid (*e-*PLA) mats obtained through two techniques: (i) direct incorporation of active compound during the electrospinning process (*e-*PLA-CIN); and (ii) supercritical carbon dioxide (scCO_2_) impregnation of CIN within electrospun PLA mats (*e-*PLA/CIN_imp_). The development and characterization of both of these active electrospun mats were investigated with the main purpose of modifying the release kinetic of this active compound. Morphological, structural, and thermal properties of these materials were also studied, and control mats *e-*PLA and *e-*${\mathrm{PLA}}_{{\mathrm{CO}}_{2}}$ were developed in order to understand the effect of electrospinning and scCO_2_ impregnation, respectively, on PLA properties. Both strategies of incorporation of this active compound into PLA matrix resulted in different morphologies that influenced chemical and physical properties of these composites and in different release kinetics of CIN. The electrospinning and scCO_2_ impregnation processes and the presence of CIN altered PLA thermal and structural properties when compared to an extruded PLA material. The incorporation of CIN through scCO_2_ impregnation resulted in higher release rate and lower diffusion coefficients when compared to active electrospun mats with CIN incorporated during the electrospinning process.

## 1. Introduction

Nanomaterials and their composites are recognized as optimal candidates for several applications because of their reduced dimensions and the effect of enhanced surface properties that provide better interfaces and chemical reaction rates [1,2]. Within these nanomaterials, it is possible to find nanofibers, nanotubes, nanorods, and nanowires, which have been researched due to their special physical and chemical properties [3]. Specifically, the nanofibers are characterized by offering unique physical, mechanical, and electrical properties associated with their very high surface area, light weight, and small pores [4]. These nanofibers can be produced by many methods, such as vapor-phase methods, solution-liquid-solid methods, template-directed methods, and self-assembly and hydrothermal synthesis methods [3], but compared to these above methods, electrospinning is characterized as a remarkably simple, low-cost, and environment-friendly technique. A typical electrospinning system consists of a high-voltage power supply, a syringe pump with a metal needle, and a grounded collector. An electric field is applied between the needle tip and the grounded collector and distorts the hemispherical surface of a droplet into a conical shape through the action of electrostatic forces. When the applied electrical force overcomes the critical surface tension of the polymer liquid, an electrically charged jet of the polymer is ejected from the tip of the Taylor cone, stretched, and finally deposited on the collector as a randomly oriented nonwoven mat of fibers ranging from micrometers to nanometers in diameter. Moreover, electrospinning is an industry-viable process that allows us to obtain a high ratio of length/diameter in a continuous process with controllable morphology and components [5,6].

Poly lactic acid (PLA) is a synthetic thermoplastic biopolymer that has attracted considerable attention in biomedical and packaging applications owing to its excellent biodegradability and non-toxicity [7,8]. PLA nanofibers have been obtained by several methods highlighting the electrospinning method [9]. Electrospun PLA nanofibers have been characterized structurally and mechanically, and the effect of the use of different solvents on their morphology and diameter has been also studied for biomedical applications [10,11,12]. Although PLA nanofibers have been designed with different purposes, over the last few years, the encapsulation of sensitive bioactive compounds has attracted special attention [13,14].

During the last few years, micro- and nano-encapsulation has been regarded as an attractive method to entrap bioactive compounds within a polymer material for the purpose of protecting and delivering active compounds at the right time and to a targeted site [15]. Various techniques have been developed to encapsulate active compounds, such as spray drying [16], freeze drying [17], emulsification [18], inclusion complexation [19], and nano-precipitation [20], but recently, electrospinning has been proposed as a feasible route to encapsulate active compounds because it is a straightforward, facile, and versatile method to produce fibers with a high surface-to-volume ratio and high porosity. Compared to the traditional encapsulation techniques, the key advantage of the electrospinning process is the absence of heat, which is important for preserving the structure and achieving high encapsulation efficacy of the active substances upon processing storage [21,22]. Specifically, nanostructured systems containing essential oils (EOs) have gained a lot of attention. EOs and their active compounds have already been used as food additives in food packaging and the cosmetic industry [23]. Cinnamaldehyde (CIN) is a biologically active compound present in the essential oil of the genus *Cinnamomun*, which is responsible for the distinctive aroma and flavor of cinnamon [24,25]. This compound is categorized as a GRAS (Generally Recognized as Safe) substance by the U.S. Food and Drug Administration and it has been already recognized due to its antioxidant and antimicrobial activity against both Gram-positive and Gram-negative bacteria, including organisms that are of concern for food safety [26,27,28]. Rieger and Schiffman have already successfully incorporated cinnamaldehyde into chitosan/poly (ethylene oxide) nanofiber mats through electrospinning for antibacterial applications [29].

Nevertheless, although the electrospinning method is relatively convenient and versatile, difficulties may be encountered in aspects of the encapsulation of sensitive bioactive compounds into fibers. The major disadvantage of conventional electrospinning is that the blend formulations often give rise to a burst release of some encapsulated compounds due to the deposition of the active components on or near the surface of the fibers [30,31]. Thus, the main objective of this study was to slow down the release kinetic of CIN, as a model of an active component, by modifying the incorporation method of this chemical compound within electrospun nanofibers. In this respect, supercritical fluid impregnation has arisen as an innovative alternative. Supercritical impregnation has been cataloged as a green process and it is basically the reverse process to supercritical extraction, where a substance is dissolved in a supercritical fluid and the high diffusivity and low surface tension of the fluid allows the polymer to swell and deposit or promote the absorption of a compound within a polymeric matrix [32,33]. Carbon dioxide is one of the most interesting supercritical fluids due to its high solvent capacity, low cost, non-toxicity, and non-flammability [34]. Villegas and coworkers have already incorporated CIN into biopolymer-based films by supercritical impregnation to develop active food packaging materials [25]. Nevertheless, the incorporation of CIN into electrospun polymeric mats and the study of its kinetic release have never been developed. In this context, this work was focused on the combination of both techniques, electrospinning and supercritical impregnation, with the purpose of developing active mats. The development of PLA nanofibers through electrospinning followed by CIN incorporation through supercritical impregnation was carried out. The PLA nanofibers’ structural and thermal properties and the CIN release kinetic assays were studied and compared with active PLA mats obtained directly through electrospinning.

## 2. Materials and Methods

### 2.1. Materials

Poly (lactic acid) (PLA), 2003D (specific gravity ¼ 1.24; Melt Flow Rate, MFR, g/10 min (210 °C, 2.16 kg)), was purchased in pellet form from Natureworks^®^ Co. (Minnetonka, MN, USA). Cinnamaldehyde (CIN) (≥99.5%) was supplied by Aldrich^®^ Chemistry (St. Louis, MO, USA). Chloroform, methanol, ethanol, dimethyl formamide (DMF), and acetonitrile of HPLC grade were supplied by Merck. Carbon dioxide was supplied by Linde (Santiago, Chile).

### 2.2. Electrospinning of e-PLA and e-PLACIN Nanofibers

The PLA fibers were obtained according to the methodology described by Arrieta et al. (2015) with some modifications [35] using an electrospinning system (Spraybase^®^power Supply Unit, Maynooth, Ireland). The effect of parameters on the electrospinning process, such as solvent effect, PLA concentration, flow rate, and working distance, were studied (shown in Supplementary Material 2S.1). Two grams of poly (lactic acid) (PLA) was added to 20 mL chloroform (CH_3_Cl):DMF (1:1) and stirred at 20 °C until the polymer was dissolved. Solutions were transferred to 5 mL plastic syringes and connected through a PTFE (polytetrafluoroethylene) tube to an 18-gauge blunt stainless steel needle charged by a high-voltage power supply with a range of 0–30 kV. The collector plate was fixed at a working distance of 14 cm below the needle tip and connected to the grounded counter electrode of the power supply. A voltage of approximately 10 kV and a flow rate of 2 mL/h were used. Electrospun PLA mat was obtained after one hour of exposure and this sample was named *e-*PLA.

In order to compare active PLA mats obtained through supercritical impregnation of CIN with active CIN-containing mats obtained through electrospinning, active *e-*PLA nanofibers were obtained by electrospinning at the same conditions a PLA solution incorporating CIN at 5% (*w*/*w*) with respect to the PLA. This active electrospun mat was named *e*-PLACIN.

### 2.3. Supercritical Fluid Impregnation of CIN in e-PLA Mats

**Figure 1:**
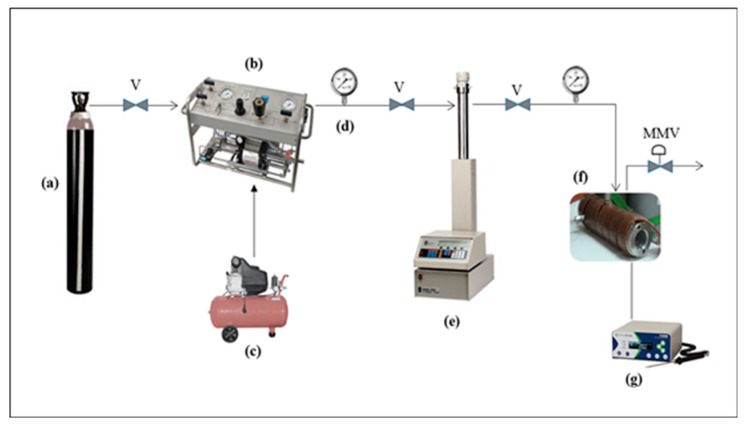
Outline of the experimental setup for the supercritical impregnation process: (**a**) Temperature controller; (**b**) High-pressure stainless steel impregnation cell; (**c**) Pressure transducer; (**d**) Syringe pump; (**e**) Air-driven pump; (**f**) Air compressor; (**g**) CO_2_ reservoir, (V) Valves, (MMV) Micrometering valve.

*e-*PLA/CIN_imp_ was obtained through supercritical fluid impregnation using the apparatus schematically described in Figure 1. This impregnation process was carried out in a high-pressure cell with a volume of 100 mL.

CIN100 µL of CIN was loaded in the cell in a 5 mL flask in order to obtain saturation in the dense supercritical carbon dioxide (scCO_2_) phase. Subsequently, *e-*PLA mats with a surface area of 152.1 ± 1.8 cm^2^ were placed into the cell and separated by a metal support, which was used to avoid direct contact between them and ensure a homogeneous impregnation for both sides. The temperature of the high-pressure cell was controlled using a thermostatic electric resistance around the cell. scCO_2_ was loaded in the system by means of an ISCO 500D syringe pump, which was operated in a constant pressure regime during the impregnation runs. Impregnation experiments were repeated for each migration experiment in turns to verify the reproducibility and reliability of this procedure. Supercritical impregnation runs were done at 12 MPa and at a constant temperature of 40 °C during 3 h. Subsequently, the cell was depressurized at 1 MPa/min and the plastic films were recovered for characterization and migrations tests. To study the effect of scCO_2_ conditions on polymer properties, a sample was exposed to the same conditions as the impregnation process without CIN, and it was named *e*-${\mathrm{PLA}}_{{\mathrm{CO}}_{2}}$.

### 2.4. Characterization of Active Electrospun PLA Mats

#### 2.4.1. Quantification of CIN in e-PLA Mats

The effective concentrations of CIN in the *e-*PLA-CIN and *e-*PLA/CIN_imp_ mats were determined immediately after each obtaining process. The analysis was performed using a method of dissolution and precipitation of the modified polymer [36] followed by a detection and quantification of the active compound carried out through high-performance liquid chromatography.

An amount of 0.2 g of each mat was dissolved into a centrifuge tube with 20 mL of chloroform at room temperature. After that, 30 mL of methanol were added to produce the precipitation of the polymer. Subsequently, the polymer was insolated by centrifugation (4500 rpm for 10 min) and the liquid phase was analyzed by high-performance liquid chromatography (HPLC). Chromatographic analysis was performed in a Hitachi LaChrom Elite HPLC (Dallas, TX, USA) equipped with a Hitachi L-2455 diode array detector and a Hitachi L-2200 autosampler. The chromatographic column used was an Inertsil ODS-3 C18, 5 μm, 4.6 mm × 250 mm. The mobile phase consisted of a mixture of acetonitrile and distilled water (40:60) at a flow rate of 2 mL min^−1^ with an injection volume of 5 μL. The oven temperature was constant at 40 °C. The detection of CIN was performed at 275 nm.

#### 2.4.2. Scanning Electronic Microscopy (SEM) Analysis

The morphologies of the electrospun mats *e-*PLA*, e*-${\mathrm{PLA}}_{{\mathrm{CO}}_{2}}$, *e-*PLA-CIN, and *e-*PLA/CIN_imp_ were studied using a scanning electron microscope (SEM) JSM-5410 Jeol (Tokyo, Japan) with the accelerating voltage at 10 kV. Samples were coated with gold palladium using a Sputtering System Hummer 6.2, and SEM micrographs of the surface were taken. Average *e*-PLA fiber diameters were analyzed with image analyzer software (Image J v1.37) (Bethesda, MD, USA).

#### 2.4.3. Fourier Transform Infrared (FTIR)–Attenuated Total Reflectance (ATR) Spectroscopy

FTIR-ATR spectroscopy was used to characterize the presence of specific chemical groups in the materials. FTIR spectra were performed in ATR mode with a Bruker IFS 66V spectrometer. The spectra were the results of 64 co-added interferograms at 4 cm^−1^ and resolutions in the wavenumber range from 4000 to 400 cm^−1^. The spectra analyses were performed using OPUS Software Version 7 (Ettlingen, Karlsruhe, Germany).

#### 2.4.4. Thermal Properties

Thermogravimetric analyses (TGA) of active PLA electrospun fibers and the PLA electrospun fibers control were carried out using a Mettler Toledo Gas Controller GC20 Stare System TGA/DCS (Schwerzenbach, Switzerland). Samples (ca. 9 mg) were heated from 20 to 600 °C at 10 °C min^−1^ under a nitrogen atmosphere (flow rate 50 mL min^−1^).

Differential Scanning Calorimetry (DSC) analyses were also performed with a Mettler Toledo DSC-822e calorimeter (Schwerzenbach, Switzerland) according to ASTM D1238. Commercial samples of indium (99.999% purity) with a melting point of *T_m_* = 156.68 °C and a melting enthalpy of Δ*H_m_* = 38.4 J g^−1^ were used as a calibration standard. An amount of 8–10 mg of PLA sample weight was heated in scanning from 25 to 200 °C at a rate of 10 °C min^−1^. All experiments were done under a purge of dry nitrogen. Glass transition (*T_g_*), cold crystallization (*T_cc_*), and melting (*T_m_*) temperatures, as well as the cold crystallization (Δ*H_cc_*) and melting (Δ*H_m_*) enthalpies, were determined from the second heating process. The degree of crystallinity (*X_c_*) of the materials was deduced from the DSC data using the following equation:

*X_c_* = % crystallinity of PLA = 100 × [(Δ*H_m_* − Δ*H_cc_*)/Δ*H*^0^*_m_*] (1)

where Δ*H_m_* is the specific melting enthalpy of the sample (J g^−1^); Δ*H_cc_* is the specific cold crystallization enthalpy of the sample (J g^−1^); and Δ*H*^0^*_m_* is the specific melting enthalpy of a wholly crystalline PLA (93.6 J g^−1^) [37].

### 2.5. Study of the Release Kinetic of CIN from Active e-PLA Electrospun Mats

#### 2.5.1. Experimental Procedure for CIN Release Rate Quantification in *e*-PLA Mats

The release of the active compound CIN from *e-*PLA-CIN and *e-*PLA/CIN_imp_ was carried out by immersion of the developed material into a food simulant following European Regulations: simulant D1 (50% *w*/*w* ethanolic solution) as a lipophilic food simulant. The release experiments were conducted at 40 °C. Double-sided, total immersion release tests were performed as follows: a 3 cm^2^ piece of each sample and 5 mL of simulant (with an area-to-volume ratio of 6 dm^2^/L) were placed in a glass vial [38]. Samples (1 mL) were periodically collected and analyzed by HPLC in order to quantify the CIN concentration in the simulant solution as a function of time. Chromatographic analyses were carried out following the same methodology explained in Section 2.4.1.

### 2.6. Statistical Analysis

A randomized experimental design was considered for the experiments. Data analysis was carried out using Statgraphics Plus 5.1 (StatPoint Inc., Herndon, VA, USA). This software was used to implement variance analysis and Fisher’s LSD test. Differences were considered significant at *p* < 0.05.

## 3. Results and Discussion

### 3.1. Incorporation of CIN in e-PLA Mats by the scCO_2_ Impregnation Process

Impregnated *e-*PLA mats were prepared by supercritical impregnation using scCO_2_ at 12 MPa, 40 °C, and a depressurization rate of 1 MPa min^−1^ during 3 h of impregnation. These process conditions were selected based on CIN’s reported solubility in scCO_2_ [39] with the aim to operate under complete miscibility between both components. Previous works in our lab have already shown that the conditions used in this work were the optimal conditions to achieve the highest impregnation rate of CIN. As Table 1 shows, the cinnamaldehyde incorporation percentage through scCO_2_ impregnation in the *e-*PLA mats reached a value of (3.29 ± 0.26)% (CIN weight/polymer weight), a lower value compared to the value obtained by Villegas et al. for extruded PLA films through a similar scCO_2_ impregnation process [25]. In that study, the incorporation percentage of CIN reached values of up to (13 ± 4)% (*w*/*w*). Although similar operational conditions were used, a CO_2_ phase was maintained saturated with CIN using an excess of this active compound (1 mL), 10 times higher than in this study, establishing a two-phase behavior along the impregnation runs. This condition generated a constant CIN concentration in the CO_2_ phase that was certainly positive for its partition towards the PLA film. Meanwhile, in this study, the CIN concentration in the CO_2_ phase decreased as the impregnation process progressed, being negative for its partition coefficient and its absorption into the *e-*PLA mat structure through the formation of hydrogen bonds between the hydrogen of the hydroxyl groups of PLA and the oxygen of the aldehyde belonging to the CIN. This interaction has been previously identified as responsible for the great affinity between PLA and CIN and also explains the higher incorporation percentage of this active compound in PLA-based materials than that obtained in other polymers, such as starch films, by means of the scCO_2_-assisted impregnation process [40].

On the other hand, the quantification of *e-*PLA-CIN mats obtained from the electrospinning process of a PLA solution with 5% CIN resulted in lower values, presenting a final percentage of (1.78 ± 0.03)% (*w*/*w*). Since electrospinning is based on a solvent evaporation process thanks to the application of an electrical field, losses of this compound due to the enhanced partial evaporation of the initial concentration of this natural volatile were expected.

### 3.2. Morphological Results of e-PLA Mats

**Figure 2:**
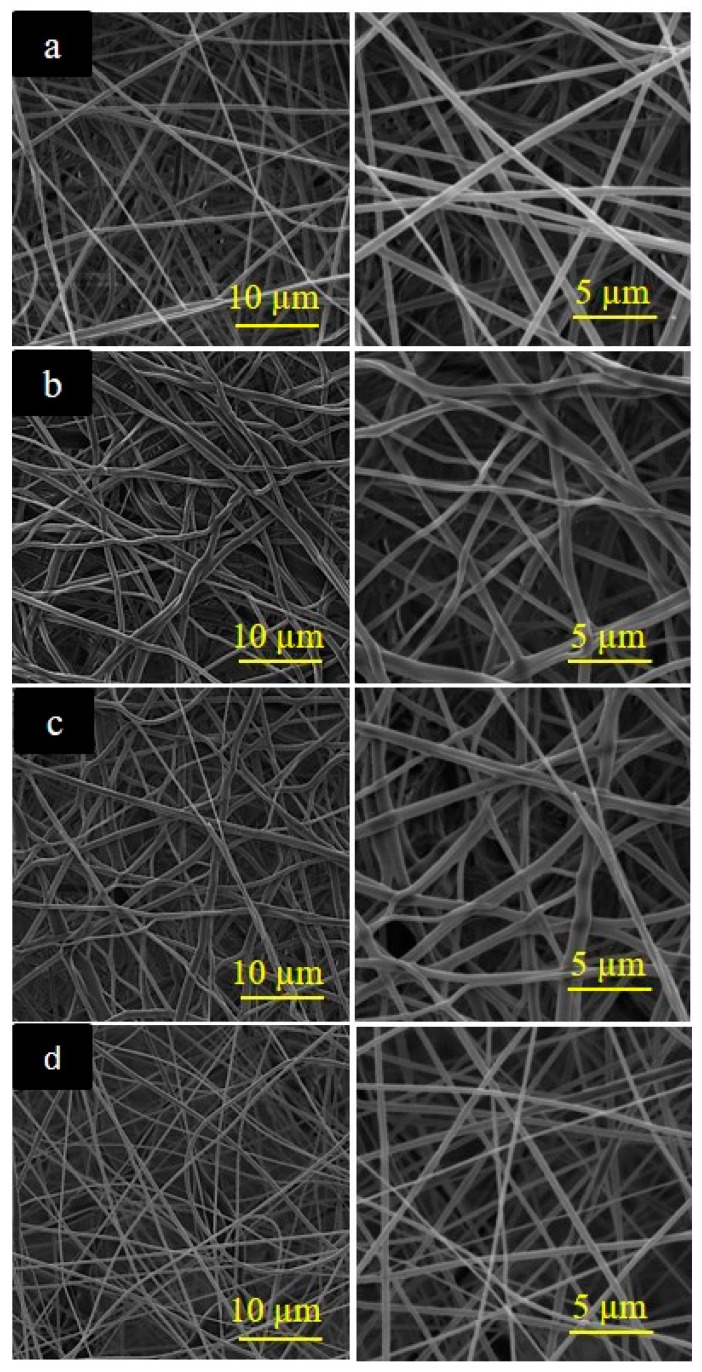
SEM micrographs at 5 and 10kx and fiber diameter distribution of electrospun PLA mats: (**a**) *e*-PLA; (**b**) *e*-${\mathrm{PLA}}_{{\mathrm{CO}}_{2}}$; (**c**) *e-*PLA/CIN_imp_; and (**d**) *e*-PLA-CIN.

**Figure 3:**
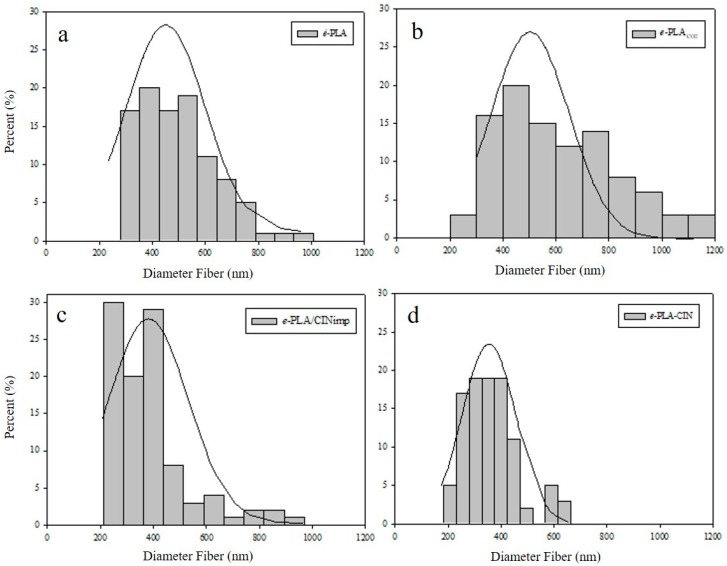
Fiber diameter distribution of electrospun PLA mats: (**a**) *e*-PLA; (**b**) *e*-${\mathrm{PLA}}_{{\mathrm{CO}}_{2}}$; (**c**) *e-*PLA/CIN_imp_; and (**d**) *e*-PLA-CIN.

SEM microscopies of the electrospun nanofibers are depicted in Figure 2 and the average fiber diameters (*n* = 50) for the electrospun PLA mats are displayed in Figure 3. Both PLA polymeric solutions (with and without CIN, Figure 2a,d, respectively) rendered continuous fibers without the presence of beads. The incorporation of CIN during the electrospinning process did not cause detectable changes in fiber morphology, and, as Figure 3 shows, *e-*PLA-CIN fibers were thinner than those of *e-*PLA. Probably, the addition of CIN decreased the viscosity of the solution, and the molecular entanglement among the components and the stretching properties of the solution resulted in the decrease of electrospun fiber diameters.

Figure 2b,c show the SEM images of electrospun PLA fibers post supercritical CO_2_ exposure, and it is possible to observe that the scCO_2_ impregnation process affected considerably the morphology of electrospun nanofibers and increased the variability of the fiber diameter range (Figure 3b,c). The microstructure of the fibers changed due to the CO_2_ pressure applied. The unique work based on scCO_2_ impregnation without active compounds on electrospun fibers called this effect “structural relaxation” [41]. In the case of *e-*${\mathrm{PLA}}_{{\mathrm{CO}}_{2}}$, there was a notable enhancement in fiber diameter and the variability of fiber diameters was high as Figure 3 shows.

### 3.3. FTIR Analysis Results

**Figure 4:**
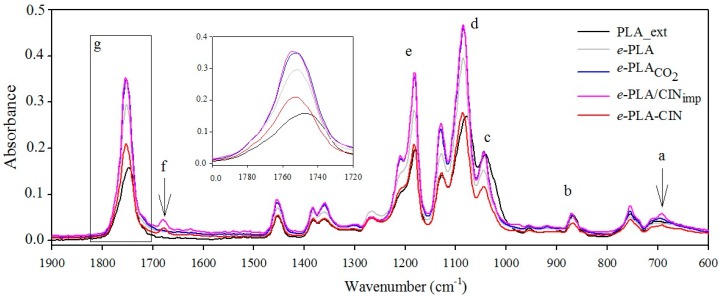
FTIR spectra of PLA materials: extruded PLA film (**black**); electrospun PLA mat, *e-*PLA (**grey**); electrospun PLA mat after scCO_2_ impregnation conditions, *e-*${\mathrm{PLA}}_{{\mathrm{CO}}_{2}}$ (**blue**); PLA mat impregnated with CIN, *e-*PLA/CIN_imp_ (**pink**); and electrospun PLA with CIN, *e-*PLA-CIN (**red**). Insert: Zoom at zone 1800–1720 cm^−1^ of PLA-based materials.

Information on the nature of the molecular interactions between CIN and the electrospun PLA polymeric matrix incorporated through both processes was monitored using infrared spectroscopy. The effect of the electrospinning process on PLA FTIR spectra was also evaluated by comparison with an extruded PLA material. Thus, FTIR spectra for extruded PLA, *e*-PLA, *e*-${\mathrm{PLA}}_{{\mathrm{CO}}_{2}}$, *e*-PLA/CIN_imp_, and *e*-PLA-CIN mats are shown in Figure 4, and Table 2 summarizes the assignments of the principal characteristic PLA and CIN bands found in the studied materials [25,42,43]. Band displacements, changes in intensity, or broadening of signals can indicate specific interactions between the components.

All spectra exhibited some characteristic peaks related to the typical absorptions of the PLA polymer, but when compared to extruded PLA material, electrospun PLA mats showed several slight displacements principally regarding those bands corresponding to C=O and C-O symmetric and asymmetric stretching. Table 2 shows the differences in wavenumber band absorptions between samples. Specifically, the carbonyl vibration of PLA when extruded showed its maximum at 1747 cm^−1^, while the electrospun mats and also the impregnated samples showed peak shifts towards higher wavenumbers (shown in Table 2), and this enhancement increased with both effects impregnation and CIN incorporation. These displacements can be clearly associated to changes in the crystallization behavior and morphology as was already observed through SEM micrographies. These band displacements were surely due to the electrospinning process and interactions between PLA chains with scCO_2_ and functional groups of cinnamaldehyde. Besides the displacement of peaks, the samples that suffered scCO_2_ impregnation also presented new peaks at 1209 cm^−1^ and 920 cm^−1^ that were associated to alkyl-ketone chain vibration and flexural C-H bond vibration, respectively. Surprisingly, these absorption bands are representative of the crystalline structure of PLA [43]. The scCO_2_ impregnation involved in the processing seems to induce the rearrangement of the chain polymer into a crystalline structure.

On the other hand, both active materials presented new characteristic bands near 1600–1700 cm^−1^ that were attributed to the vibrations of the aromatic ring and to the aldehyde group of CIN [40], and a new peak at 691 cm^−1^ corresponding to the phenyl group of CIN, specifically the CH=CH bending out-of-plane in alkenes. As Table 2 shows, the incorporation of CIN within an electrospun PLA mat through electrospinning resulted in a lower displacement of peak frequency, indicating that the encapsulation process was more a physical incorporation than a chemical interaction [44].

### 3.4. Thermal Characterization of e-PLA Mats

**Figure 5:**
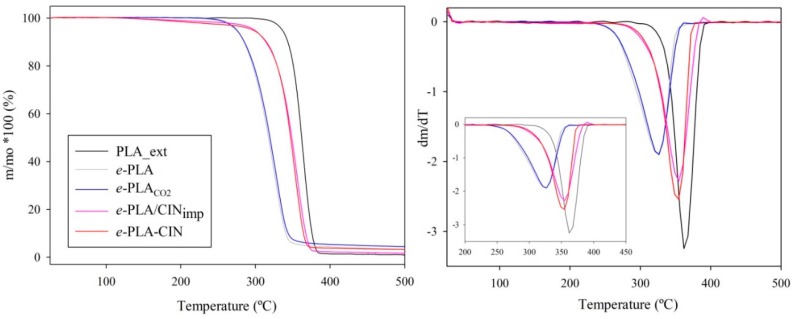
(**Left**) TGA curves of PLA materials. (**Right**) Derivative plots of TGA (DTGA) of PLA-based materials. Insert: Zoom between 200 °C and 450 °C.

Thermal analyses of electrospun mats were performed to study the effect of the impregnation process, and the incorporation of the active agent, on the thermal stability and morphology of the polymer. Moreover, a sample of extruded PLA was also analyzed in order to observe the influence of the electrospinning process. Figure 5 shows the weight loss and the derivative of the weight loss with temperature. All PLA-based materials presented a single band of degradation. As Figure 5 shows and Table 3 indicates, the electrospinning process decreased the thermal stability of the PLA polymer. The maximum degradation of all *e*-PLA mats occurred at lower temperatures than those of the extruded PLA. This fact was already seen in previous works based on polyvinyl alcohol nanofibers [45]. The electrospinning process induced a change in the polymer nanoscale structure that involved an increase in specific area, and, hence, the heat penetrated faster. Regarding scCO_2_ impregnation, although the morphology of the nanofibers was altered (shown in Figure 2), this process did not present an important effect.

As was expected due to the antioxidant character of this compound, the presence of CIN increased the polymer’s thermal stability at a similar rate when incorporated by both methods [28].

Table 3 summarizes the main thermal properties of PLA mats obtained from DSC thermograms compared also with an extruded PLA material. As can be seen, the type of processing resulted in a significant change in the thermal properties of PLA materials. As the glass transition temperature (*T_g_*) values show, electrospinning resulted in a more plasticized material and this effect was enhanced by the incorporation of CIN between the polymer chains, which increased their mobility. Although mats were rigorously dried under vacuum before analysis, the presence of residual solvent from the electrospinning process decreased the intermolecular and intramolecular interaction between the polymeric chains. Lopez de Dicastillo et al. have already observed this feature in a previous study based on the comparison of materials obtained through casting (dissolution-evaporation) with extruded polymers [46]. Regarding the effect of CIN, this plasticizing effect is common with the incorporation of essential oils and other active components, such as ascorbic acid, α-tocopherol, butylated hydroxytoluene (BHT), and marigold flower extract, into polymeric matrixes and results in a more flexible and ductile material [46,47,48]. Moreover, the lower chemical interaction between PLA groups with CIN in the case of *e*-PLACIN mats induced the highest decrease in these values. This plasticizing effect and increase in PLA chains’ mobility also turned into a great decrease of *T_cc_* values caused by the incorporation of CIN between polymeric chains, which promoted the crystallization of PLA in less stable α’crystals at lower temperatures [49]. *e*-PLA mats also presented a lower melting temperature compared to the extruded PLA material. All of these experimental results pointed to a decreased crystalline structure.

*X_c_*’ values were also clearly influenced by the electrospinning process and incorporation of CIN. The electrospinning process decreased PLA crystallinity. Probably, the fast solvent evaporation (chloroform and DMF) during the electrospinning process implied the generation of a lower rate of PLA crystals. On the other hand, the presence of CIN increased the formation of PLA crystals, principally in the case of *e*-PLACIN.

The incorporation of CIN during the electrospinning process induced the rearrangement of the chains and showed some nucleating effect. Other works have already mentioned that the influence of active compounds on the *X_c_*’ values depends on the compound’s nature [50,51].

### 3.5. Study of the Release Kinetic of CIN from e-PLA-CIN and e-PLA/CIN_imp_ Mats

**Figure 6:**
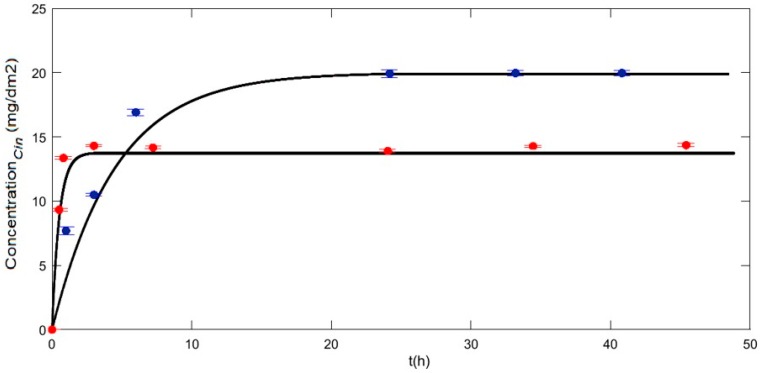
Release kinetic of cinnamaldehyde from *e-*PLA/CIN_imp_ (**blue dots**) and *e-*PLA-CIN (**red dots**) in EtOH 50%, as a food simulant, at 40 °C.

The mass transfer during CIN release from *e*-PLA mats was experimentally studied by means of the specific migration tests previously described in Section 2.5.1 Experimental release kinetics of CIN from *e-*PLA/CIN_imp_ and *e-*PLA-CIN mats were carried out using 50% (*w*/*w*) ethanolic solution, as a fatty food simulant, at 40 °C. These tests allowed for us to observe the dependence of the CIN release on the type of active compound incorporation process. Figure 6 shows the CIN concentration change as a function of time obtained from the release experiments as well as the theoretical curves generated by means of the transfer model with the correlated diffusion coefficient of CIN in *e*-PLA-CIN and *e-*PLA/CIN_imp_ mats.

These experiments were conducted until a plateau of CIN concentration in the solutions as a function of time was achieved. Thus, the last concentration registered was considered to be measured under an equilibrium condition; meanwhile, the instantaneous average active compound concentration value in the polymer film was estimated by mass balance from its initial concentration value. After the estimation of the CIN equilibrium concentrations in the sample and in the simulant solution (*SS*), the partition coefficient of CIN between the polymer phase and the simulant solution, *K_PLA/SS_*, was calculated and subsequently used as input data in the mathematical model [23].

#### Determination of Partition and Diffusion Coefficients of CIN in *e*-PLA Mats

The mass transfer description of the migration process through *e*-PLA-CIN and *e-*PLA/CIN_imp_ can be achieved by means of Fick’s law. Distribution coefficients between the polymer layer and the food simulant were directly calculated from the equilibrium concentration in the food simulant and the mass balance in the plastic films [52]. The mass transfer of the active compound from the PLA nanofibers mat to a liquid phase was described by a one-dimensional simplification of the Fick’s Law. In this way, the instantaneous mass transfer of CIN occurs in a symmetrical process on both sides of the plastic film, which is completely immersed in the receiving solution. In this way, the symmetrical transfer rate of CIN through the polymer film can be quantified by the following Equation (2):

(2) $$J_{I}=\;\frac{D_{Cin}}{L/2}\cdot \left(C_{Cin}^{PLA\;}\left(x=0,t\right)-C_{Cin}^{PLA}\left(x=L/2,t\right)\right)$$

where $J_{I}$ is the mass transfer flux (kg m^−2^ s^−1^) of CIN through the impregnated and electrospun PLA nanofibers mat, $D_{Cin}$ (m^−2^ s^−1^) is the diffusion coefficient of CIN in the polymer, *L* represents the film thickness (m), and $C_{Cin}^{PLA}$ is the highest concentration value of CIN (kg m^−3^) in the middle of the polymer thickness (*x* = 0) and in the polymer interface in contact with the receiving phase (*x* = *L*/2).

The thermodynamic equilibrium established at the polymer–solution interface can be quantified by the partition coefficient *K_PLA/SS_*. This parameter was calculated according to Equation (3):

(3) $$K_{PLA/SS}=\;\frac{C_{Cin}^{PLA}\;\left(x=L/2,t\right)}{C_{Cin}^{SS}\;\left(x=L/2,t\right)}.$$

The last transfer step in the CIN release is represented by the transfer through the boundary layer of the solution simulant phase, which can be described by Equation (4):

(4) $$J_{II}=k\cdot \;\left(C_{Cin}^{SS}\left(x=L/2,t\right)-C_{Cin}^{SS}\left(x=\infty ,t\right)\right)$$

where $C_{Cin}^{SS}$ is the concentration of CIN (kg m^−3^) at the interface (*x* = *L*/2) and in the bulk of the simulant solution (*x* = ∞). Meanwhile, *k* (m s^−1^) represents the mass transfer coefficient under natural convection transport in the solution and its value was calculated by means of the correlation reported by Galotto and coworkers [53] where the coefficient is obtained from the Sherwood number, which is calculated as a function of the Grashof and Schmidt numbers.

The equation system can be solved considering the initial conditions and other assumptions related to the interactions between the polymer and the receiving solution. These considerations are listed below: (1) The initial concentration of CIN in the impregnated PLA nanofibers mat is known and homogeneous in the whole phase; (2) The simulant solution is initially CIN-free and no mass transfer limitations are considered in the solution. Thus, the active compound is considered to be homogeneously distributed in the whole receiving phase; and (3) Physicochemical interactions between the PLA nanofibers mat and the receiving phase are considered negligible. In this way, these phases are considered immiscible. The mass transfer equations were solved under a steady-state condition for an instantaneous time. The Regula Falsi algorithm was applied to reduce the number of iterative calculations, which have been achieved by means of a script developed in MatLab^®^ according to a routine that considers the income of the structural parameters of the system and the initial experimental conditions in which migration testing is performed. Thus, concentrations of CIN in the bulk of the polymer and in the simulant solution were recalculated after each time step by mass balance using the instantaneous value of the CIN transfer flux.

The value of *K_PLA/SS_* calculated from Equation (3) was used as the input parameter, which was fed into the model. Thus, the mathematical model was used to simulate different release kinetics using different values for the diffusion coefficient of CIN in a PLA nanofibers mat, *D_Cin_*. The chosen value of the diffusion coefficient is that which shows the closest values to the simulated and experimental release kinetics. This procedure was conducted from the lowest values of the root of the mean square error (RMSE) between the experimental data and predicted values of CIN concentration in the simulants as a function of time [54]. *RMSE* was calculated as follows:

(5) $$RMSE=\frac{1}{M_{P,0}}\cdot \sqrt{\left(\frac{1}{N}\right)\cdot \sum_{t=1}^{N}{\left({\left(M_{SS,t}\right)}_{\exp erimental,t}-{\left(M_{SS,t}\right)}_{predicted,t}\right)}^{2}}$$

where *N* is the experimental points number for each migration curve, *i* is the observations number, *M_P_*_,0_ is the initial amount of the active compound in the polymer (µg), and *M_SS_*_,*t*_ is the active compound amount in the simulant at time *t* (µg).

Thus, at equilibrium, the remaining concentration of CIN in *e-*PLA mats was quantified and the partition coefficients of CIN, *K_PLA/SS_*, were calculated according to Equation (3). This coefficient compares the relative affinity of the active compound between the polymeric phase (*e-*PLA) and the liquid phase (50% ethanolic solution) [23]. As Table 4 shows, the lowest value of *K_PLA/SS_* was obtained for the impregnated PLA films, indicating that the higher affinity of the active compound was for the food simulant phase rather than for the polymeric phase. The main reason is because scCO_2_ was able to diffuse into the polymeric mat and swelled it, which favored both the incorporation of active substances previously dissolved and the release afterwards. In this way, solute loading was not limited to the surface, but it was also located deeper inside the polymeric matrix [55]. In general, this behavior of swelling and/or plasticizing of the polymer favored additive incorporation and yielded higher loadings and a more uniform distribution of the additives or compounds in the polymer phase [56].

Once *K_PLA/SS_* was obtained for the CIN release process, the mathematical model previously developed was used to simulate the release kinetic correlating the diffusion coefficient of the active compound for both systems analyzed, *D_Cin_*. This value was obtained by correlation of the experimental data using the mass transfer model. In the last column of Table 4, the value of the root mean square error (RMSE) of the model solution related to the experimental data was reported for each migration test. From these results, it was possible to observe that the migration equilibrium was reached after approximately 20 h in the case of impregnated mats *e-*PLA/CIN_imp_. Meanwhile, the migration equilibrium was obtained after 1 h for the electrospun mats *e-*PLA-CIN. This behavior can be explained because the impregnation process allowed for a better chemical interaction between the CIN and the polymeric matrix mainly though the formation of hydrogen bonds between the hydrogen of PLA hydroxyl groups and the oxygen of the aldehyde belonging to the active compound. Furthermore, the changes in the polymer’s morphology that occurred due to the scCO_2_ impregnation, clearly shown though SEM microscopy, resulted in the slowdown of the release process. Calculations with the MatLab script revealed that the diffusion coefficient values of CIN strongly depended on the incorporation process of the active compound. Thus, the diffusion coefficient values were equal to 1 × 10^−12^ (m^2^ s^−1^) and 6 × 10^−^^14^ (m^2^ s^−^^1^) for the *e-*PLA-CIN and *e-*PLA/CIN_imp_ mats, respectively*.* This behavior can be explained by two main factors: (i) the high porosity (large surface area) of the fiber mats which increased the mass transfer rate [57]; and (ii) a short diffusion passage length provided by small diameters of the fibers [58]. Another reason to explain this behavior could be the plasticizing effect that caused a decrease of *T_g_* and an increase in the mobility of polymeric chains allowing for a rapid diffusion.

Generally, electrospun fibers with a lower diameter display more superficial area, which increases the distribution of the active compound. Since both fibers have similar diameters, the differences in kinetic release were only due to the changes in morphology caused by the scCO_2_ impregnation process.

On the other hand, during the preparation of the fibers through electrospinning and due to a high ionic strength, the rapid evaporation of the solvent in the mixture induced the localization of the active compound predominantly on the surface of the fiber [6]. Torres and coworkers [23] have studied the release of thymol from PLA films in a different simulant (EtOH 10–95%) and obtained diffusion coefficient values in the same order of magnitude. These authors also indicated that the greater affinity for the ethanol content in the solutions is due to the non-polar character of both thymol and the simulant solution. The release properties of tetracycline hydrochloride from poly (ethylene-covinylacetate) and poly-l-lactic acid (PLLA) electrospun mats have also been investigated by Kenawy et al. [59]. In all of the cases, a burst release occurred during the first 10–12 h. A similar phenomenon was also observed by Zong et al., where Mefoxin was found to have a high burst release from poly-d,l-lactic acid (PDLLA) electrospun fibrous mats in the first 3 h [60]. This type of release rate is characteristic for medicines and drugs because electrospinning can provide fiber carriers for drug delivery with outstanding features at diverse levels. For example, drugs can be conveniently incorporated into the carrier polymers without structural and bioactive alteration by a simple process that provides a fast mass transfer rate and an efficient drug release rate [61].

In this study, it is important to highlight that the release kinetic was strongly affected by the CIN incorporation process, and as Table 4 and Figure 6 show, important differences in the release kinetic parameters were found. These results show the possible potential use of these materials in different areas, such as food packaging or the pharmaceutical and medical industries.

## Figures and Tables

**Table 1:** Fiber diameter of electrospun poly lactic acid (*e-*PLA) mats and cinnamaldehyde (CIN) content of active materials.

*e-*Mats	Fiber Diameter (nm)	CIN Content (%)
*e*-PLA	495 ± 147	0
*e*-${\mathrm{PLA}}_{{\mathrm{CO}}_{2}}$	609 ± 218	0
*e-*PLA/CIN_imp_	384 ± 149	3.29 ± 0.26
*e-*PLA-CIN	362 ± 102	1.78 ± 0.03

*e-*${\mathrm{PLA}}_{{\mathrm{CO}}_{2}}$ = electrospun PLA mat after scCO_2_ impregnation conditions; PLA/CIN_imp_ = PLA mat impregnated with CIN; *e-*PLA-CINn = electrospun PLA with CIN.

**Table 2:** Characteristic wavenumbers expressed in (cm^-1^) assigned to FTIR absorption bands of PLA mats.

Peaks	PLA ext	*e*-PLA	*e*-${\mathrm{PLA}}_{{\mathrm{CO}}_{2}}$	*e*-PLA/CIN_imp_	*e*-PLA-CIN	Assignment
a	-	-	-	691	691	CH=CH bending in alkene of CIN
b	867	867	870	870	867	PLA amorphous zone
c	1040	1044	-	-	-	C-O stretching
d	1080	1085	-	-	-	C=O and C-O symmetric stretching
e	1180	1182	-	-	-	C-O-C stretching
f	-	-	-	1681	1679	aromatic ring and aldehyde group of CIN
g	1747	1751	1752	1754	1753	C=O carbonyl stretching

**Table 3:** Thermal properties of *e*-PLA-based mats.

Materials	*T_deg_*	*T_g_* (°C)	*T_cc_* (°C)	Δ*H_cc_* (J/g)	*T_m_* (°C)	Δ*H_m_* (J/g)	*X_c_*’ (%)
PLA ext	365.1 ± 1.5 b	63.2 ± 0.7 d	117.2 ± 0.3 b	22.3 ± 0.1 b	155.6 ± 1.7 c	26.5 ± 0.5 b	4.6 ± 0.4 b
*e*-PLA	334.0 ± 11.1 a	53.1 ± 0.2 c	113.8 ± 0.2 b	24.7 ± 4.5 b	153.2 ± 0.3 bc	25.8 ± 4.2 b	1.1 ± 0.4 a
*e*-${\mathrm{PLA}}_{{\mathrm{CO}}_{2}}$	334.6 ± 8.3 a	56.7 ± 0.1 c	122.9 ± 0.5 b	8.0 ± 1.6 a	150.9 ± 0.8 b	9.3 ± 2.3 a	1.4 ± 0.8 a
*e*-PLA/CIN_imp_	350.8 ± 8.2 ab	46.5 ± 2.7 b	103.7 ± 7.2 a	24.8 ± 0.2 b	151.2 ± 0.4 b	27.0 ± 0.4 b	2.4 ± 0.2 a
*e*-PLA-CIN	349.8 ± 5.9 ab	38.2 ± 3.9 a	100.3 ± 4.1 a	25.3 ± 0.8 b	147.3 ± 1.5 a	30.3 ± 0.2 b	5.4 ± 0.6 b

Lower case letters a–d indicate significant differences in a thermal parameter among the materials.

**Table 4:** Partition and diffusion coefficients and root mean square error (RMSE) values of cinnamaldehyde from different active mats in EtOH 50% at 40 °C.

*e-*Mats	*K_PLA/SS_*	*D_Cin_* (m^−2^ s^−1^)	*RMSE* (%)
*e-*PLACIN	470	1 × 10^−12^	0.65
*e-*PLA/CIN_imp_	133	6 × 10^−14^	0.71

## Supplementary Files

Supplementary File 1
