# Supplementary material for: Modifying an Active Compound’s Release Kinetic Using a Supercritical Impregnation Process to Incorporate an Active Agent into PLA Electrospun Mats
Source: Polymers (Basel). 2018 Apr 27;10(5):479. doi: 10.3390/polym10050479 (PMC6415505; doi:10.3390/polym10050479)
Supplement: Supplementary File 1 [file polymers-10-00479-s001.pdf]

polymers

MDPI

Article

Supplementary material

2S. Materials and Methods

2S.1. Preparation of Electrospun PLA Nanofibers

The PLA fibers were obtained according to the methodology described by Arrieta et al., 2015 with some modifications. The effect of different parameters on the electrospinning process was studied. Firstly, the search for appropriate solvent was carried out, followed by the study of PLA concentration, flow rate, and the distance between the needle and the collector (values shown in Table 1S). It is worthwhile mentioning that each parameter was estimated one after the other.

Table S1. Parameters studied on electrospinning process.

Parameters	Variables				
CH_3Cl : DMF	80 : 20	70 : 30	60 : 40	50 : 50	
PLA (%)	8	9	10		
Flow rate (mL . h^{-1})	1.25	1.5	1.75	2	
Distance (cm)	8	10	12	14	16

3S. Results and discussion

3S.1. Results of electrospinning processes

Firstly, a PLA solution at 8% (w/v) in different solutions chloroform ($CHCl_3$): N, N-dimethylformamide (DMF) was tested: (80:20), (70:30), (60:40) and (50:50). In all these cases, the needle diameter was 0.7 mm, the injection flow was set at 1.0 mL h^{-1}, and the electrospun fibers were received in a grounded aluminum foil collector located perpendicularly at 14 cm from a charged needle. The unique solvent mixture that allowed for the electrospinning process was a mixture $CHCl_3$:DMF (50:50) because the other solvent mixtures resulted in a constant formation of a crown (dry polymer stuck at the tip of the needle) and the rapid evaporation of chloroform, causing the constant dripping of the polymer. With the mixture ratio CH_3Cl:DMF (50:50), the crown was no longer, but the Taylor´s Cone was not correctly stabilized, resulting in the presence of several beads. In order to solve this instability, the PLA concentration was increased. Thus, afterwards, the polymeric concentration was varied to 9 and 10% (w/v) maintaining a needle diameter of 0.7 mm, the flow rate at 1 mL h^{-1} and the distance between the collector and the tip of the needle at 14 cm. The sample that showed the best behavior was the concentration of 10% (w/v) whose Taylor´s Cone remained stable and the number of beads considerably decreased.

Increasing the flow rate was also an important parameter to improve in order to diminish the time necessary to acquire the *e*-PLA mats.

As the *e*-PLA mats' collection time depended on the flow rate, it was also interesting to increase it as much as possible with the intention of accelerating the process. Thus, the next step was the search for the greatest flow that allowed for us to obtain homogeneous fibers. The flow rates tested were 1.25; 1.5; 1.75; and 2 mL h^{-1}. In general, all flow rates resulted in homogenous fibers with a stable Taylor´s Cone, although as the flow rate increased, the voltage necessary for the process also increased from 8.3 to 10.9 V.

Finally, the effect of the distance between the tip of the needle and the collector in the electrospinning process was evaluated and results are shown in Figure S1. The principal variation resulted in the increase of the collection area. At the lowest values of distance, the diameter of the collection area was short and fibers presented a high amount of beads and were slightly humid. The

most convenient diameter area of collection and homogenous fibers were obtained at a distance of 14 cm.

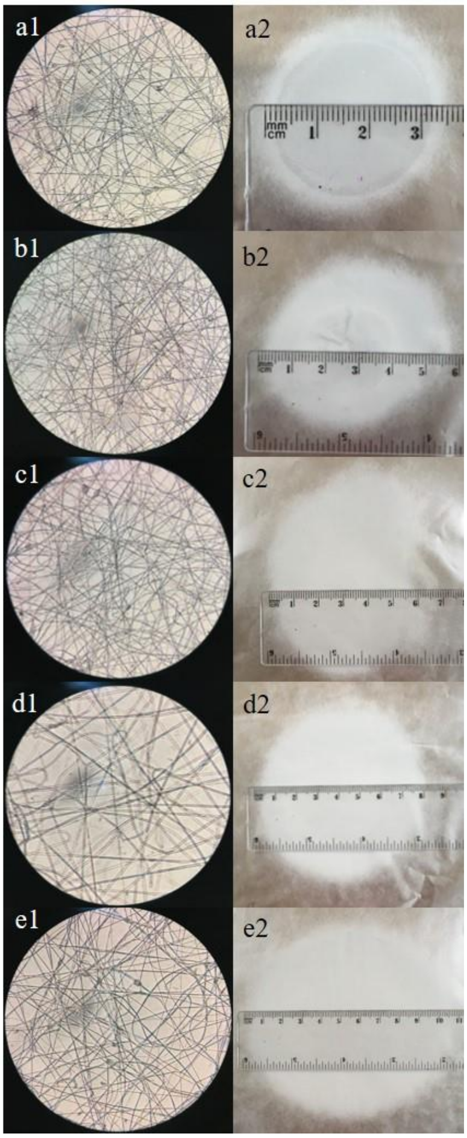

Figure S1. (1) Microscope images with a 40x magnification and (2) photographs of the *e*-PLA mats emphasizing the resulting diameter of the collection area. *e*-PLA fibers were obtained using PLA solution at 10% (w/v) in CH_3Cl:DMF (1: 1), needle 0.7 mm, flow rate 2 mL h^{-1} and different distances between the collector plate and the tip of the capillary: a) 8 cm, b) 10 cm, c) 12 cm, d) 14 cm, and e) 16 cm.

© 2018 by the authors. Submitted for possible open access publication under the terms and conditions of the Creative Commons Attribution (CC BY) license (http://creativecommons.org/licenses/by/4.0/).
